# Bending Analysis of Asymmetric Functionally Graded Material Sandwich Plates in Thermal Environments

**DOI:** 10.3390/ma16134682

**Published:** 2023-06-28

**Authors:** Mengna Han, Jiahao Huang, Zhicheng Huang, Xingguo Wang

**Affiliations:** College of Mechanical and Electronic Engineering, Jingdezhen Ceramic University, Jingdezhen 333001, China; m15214787876@163.com (M.H.); 13553414231@163.com (J.H.); wangxingguo@jci.edu.cn (X.W.)

**Keywords:** functionally graded materials, sandwich plates, thermo-mechanical bending, asymmetric, deflection and stress

## Abstract

This paper investigates the bending of asymmetric functionally graded material (FGM) sandwich plates subjected to thermo-mechanical loads in thermal environments. In this paper, a thermo-mechanical analysis model for asymmetric FGM sandwich plates is proposed, which contains only four control equations and four unknown variables. The governing equation is obtained through refined shear theory and the principle of virtual work, and the Navier method is used to solve it. Numerical examples of simply supported FGM sandwich plates under thermo-mechanical loads are given to verify the accuracy of the model. Finally, detailed studies are conducted on the bending of asymmetric FGM sandwich plates under thermo-mechanical loads, exploring the effects of various parameter changes on their bending behavior, and providing strong guidance for the application of asymmetric FGM sandwich plates in industrial production practice.

## 1. Introduction

The sandwich structure is one of the widely used composite structures in the composite industry [1]. Compared with pure composite materials, such as fiber-reinforced polymer composites and titanium alloys, it has excellent bending stiffness, low specific weight, superior vibration characteristics, and good fatigue performance [2]. Because of its excellent performance, it is widely used in aircraft, aerospace, shipbuilding, construction, transportation, and other industries. However, in the traditional sandwich structure, the core layer is sandwiched by two homogeneous material faceplates. The connection between them is mostly bonding, which leads to a discontinuity in the material properties of the surface layers and the core layer. This will lead to delamination of sandwich plates. At the same time, there will be residual stress due to the difference in temperature coefficient between different materials. In recent years, some research workers have introduced functionally graded materials (FGMs) into sandwich structures to overcome the above problems. A functionally graded material is a high-level non-uniform composite material composed of different phases, whose material properties change continuously in one or more directions [3,4]. Therefore, FGMs with continuously changing material properties can alleviate the sudden change in thermo-mechanical properties at the interface of sandwich plates, thereby realizing the application of the sandwich structure under specific requirements.

Due to the increasing demand for the use of FGMs in sandwich structures, it is necessary to understand the mechanical properties of sandwich structures containing FGMs under various conditions. In recent years, a large number of scholars have conducted extensive research on the changes in mechanical properties of FGM sandwich structures under force, thermal, and thermo-mechanical loads. Yang et al. [5] studied the nonlinear local bending of FGM sandwich plates under lateral patch loading based on the first-order shear deformation theory and von Karman nonlinear dynamics, and performed a parametric analysis of the structure. Zenkour [6] analyzed the thermo-mechanical bending of functionally graded material sandwich plates under simple support using the improved sinusoidal shear deformation plate theory. The effects of shear deformation and normal deformation were considered in this study. Daouadji et al. [7] proposed an original hyperbolic and parabolic shear and normal deformation theory considering the effect of mid-thickness tension in functionally graded sandwich plates and then studied the mechanical analysis of functionally graded sandwich plates. Zarga et al. [8] used a simple quasi-3D shear deformation theory to perform a thermal bending analysis of functionally graded material sandwich structures, and the method considered a new kind of kinematics. Rouzegar et al. [9] conducted a thermo-elastic bending analysis of ceramic-metal FG sandwich plates based on the hyperbolic shear deformation theory. They also discussed the influence of the change in influence parameters on the thermo-elastic bending of sandwich plates. Di Sciuva and Sorrenti [10] evaluated the flexural and free vibration performance of single-layer FG plates and FG sandwich plates under different boundary conditions based on a refined zig-zag theory. Li et al. [11] used the four-variable fine plate theory to study the thermo-mechanical bending of FGM sandwich plates under the simply supported boundary condition, and studied the influence of various parameters on the bending properties of sandwich plates. Trinh et al. [12] studied the deterministic and stochastic dynamics of FG sandwich plates under thermo-mechanical loads based on the third-order shear deformation theory. Liu et al. [13] used shear deformation shell theory and Hamilton’s principle to study the impact response of a sandwich cylindrical shell composed of a porous FG core. Yoosefian et al. [14] used first-order shear theory and Van Karman’s nonlinear strain-displacement relationship to investigate the nonlinear thermo-mechanical bending of circular/ring FGM sandwich plates. Daikh et al. [15] applied the high-order shear deformation theory to study the thermo-mechanical bending behavior of FG sandwich plates, and carried out parameter analysis. Naveenkumar et al. [16] studied the analytical formulations and solutions for the flexural analysis of FGM sandwich plates with the available high-order fine-grained computational models and presented numerous numerical results for in-plane, transverse displacements, and stresses. Liu et al. [17] studied the nonlinear dynamic response of porous FG sandwich cylindrical shells embedded in elastic media using modified Donnell nonlinear shell theory and Hamilton’s principle.

To reduce the difficulty of predicting the bending properties of FGM sandwich plates, scholars have made continuous improvements to the theory. Houari et al. [18] developed a new advanced shear and normal deformation theory to analyze the thermo-elastic bending of FGM sandwich plates. The theory divides the transverse displacement into bending, shearing, and thickness-stretching components. In addition, there are only five unknowns. Tounsi et al. [19] proposed an improved triangular shear deformation theory considering the effect of transverse shear deformation to analyze the bending of FG sandwich plates in a thermal environment. The number of unknown functions involved in the theory was only four, and there was no need to introduce shear correction coefficients. Kaci et al. [20] proposed an efficacious and uncomplicated refined theory to study the nonlinear bending analysis of functionally graded sandwich plates. The theory only gave four governing equations and only involved three unknown variables. Houari et al. [21] conducted a bending analysis of functionally graded sandwich plates in a thermal environment using the bivariate fine-plate theory, which involved only four variables. Abdelaziz et al. [22] used a new higher-order shear deformation theory to analyze the mechanical bending of functionally graded sandwich plates, which, unlike other theories, only involved four variables. Zenkour et al. [23] proposed an improved triangular high-order plate theory for analyzing the mechanical bending of simply supported ceramic-metal functionally graded sandwich plates. The theory took into account transverse shear strain and transverse normal strain, and the number of unknown functions involved was only four. Mantari et al. [24] used a new first-order shear deformation theory to study the simply supported functionally graded sandwich plates, which only included four unknowns. Tlidjiet al. [25] used the new four-variable fine plate theory to study the bending response of functionally graded material sandwich plates under thermo-mechanical load, and there were only four control equations and four unknown variables in this model. Bouamoud et al. [26] used a four-variable plate model to study two FGM sandwich plates under thermo-mechanical load, and this model only involved four unknowns. Finally, they carried out a detailed parameter study.

At the same time, some scholars also used the finite element method to analyze the bending properties of functionally graded material sandwich structures. Van do et al. [27] proposed an improved meshless radial point interpolation method to analyze the nonlinear bending of functionally graded plates under simply supported or clamped boundary conditions. Hirane et al. [28] proposed a fixed C^0^ high-order layered finite element model to analyze the static and free vibration of FGM sandwich plates under different boundary conditions. Naghavi et al. [29] used the finite strip method based on the refined plate theory to perform a mechanical bending analysis of two functionally graded sandwich plates under different boundary conditions. In this study, the finite strip formula was combined with refined plate theory, and the functionally graded sandwich plates were analyzed. Vinh [30] combined high-order shear deformation theory with the finite element method for the study of bi-directional functionally graded sandwich plates. Finally, the parameters were studied.

For the analysis of the mechanical performance of FGM sandwich plates, most scholars have adopted various methods to study their performance changes under force loads, thermal loads, and thermo-mechanical loads. However, in existing research, there are few studies specifically involving the mechanical properties of asymmetric FGM sandwich plates. In practical production applications, asymmetric FGM sandwich plates are also used. Compared to symmetric FGM sandwich plates, there are significant differences in the mechanical properties of asymmetric FGM sandwich plates. Therefore, it is important to study asymmetric FGM sandwich plates. This article focuses on the thermo-mechanical bending of asymmetric FGM sandwich plates. Firstly, based on the refined shear deformation theory and the shape functions of three different displacement fields, the displacement fields of FGM sandwich plates are obtained. Next, using the principle of virtual work, the control equation is obtained. Then, the Navier method is used to obtain the exact solution of the asymmetric FGM sandwich plates under simply supported boundary conditions. Finally, detailed studies are conducted on the influence of parameter changes on the thermo-mechanical bending of asymmetric FGM sandwich plates, providing strong guidance for the application of asymmetric FGM sandwich plates in industrial production practice.

## 2. Theoretical Models and Formulas

**Figure 1:**
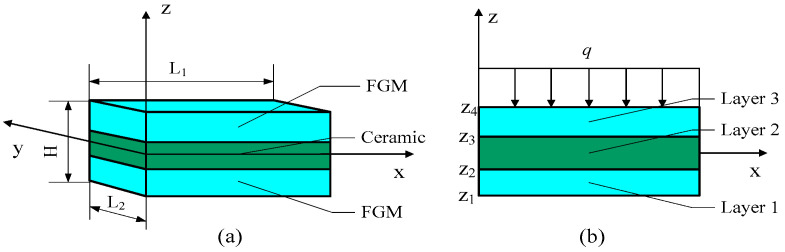
Geometry of functionally graded material sandwich rectangular plate in Cartesian coordinates. (**a**) three dimensional coordinates of FGM sandwich plate; (**b**) two dimensional coordinates of FGM sandwich plate.

In this paper, the FGM sandwich plate is composed of two FGM faceplates and a homogeneous material core layer. The core layer is a ceramic layer. The length, width, and thickness of the FGM sandwich plate are $L_{1}$, $L_{2}$, and H, respectively. The established coordinate system is shown in Figure 1. There is a transverse load q on the top surface of the FGM sandwich plate.

The material properties of FGM can be expressed by the Voigt model as [31]:

(1) $$p\left(z\right)=p_{c}V_{c}\left(z\right)+p_{m}V_{m}\left(z\right)\;$$

where $p_{c}$ and $p_{m}$ are the material properties (such as Young’s modulus, Poisson’s ratio, and thermal expansion coefficient) of ceramics and metals, respectively. $V_{c}$ and $V_{m}$ are the volume fractions of ceramic and metal, respectively, and they satisfy the relationship of $V_{c}+V_{m}=1$.

In the FGM sandwich plate, $V_{c}^{\left(i\right)}$ is expressed as:

(2) $$\left\{\begin{array}{l} V_{c}^{\left(1\right)}\left(z\right)={\left(\frac{z-z_{1}}{z_{2}-z_{1}}\right)}^{s}\;\;\;\;\;z\in \left[z_{1},z_{2}\right]\\ V_{c}^{\left(2\right)}\left(z\right)=1\;\;\;\;\;\;\;\;\;\;\;\;\;\;\;\;\;\;z\in \left[z_{2},z_{3}\right]\\ V_{c}^{\left(3\right)}\left(z\right)={\left(\frac{z-z_{4}}{z_{3}-z_{4}}\right)}^{s}\;\;\;\;\;z\in \left[z_{3},z_{4}\right] \end{array}\right.$$

where s is the volume fraction index, and s=0 represents a fully ceramic plate.

According to the refined shear deformation theory, the following displacement field can be obtained [32]:

(3) $$\left\{\begin{array}{l} u\left(x,y,z\right)=u_{1}\left(x,y\right)-z\frac{\partial w_{1}}{\partial x}-f\left(z\right)\frac{\partial w_{2}}{\partial x}\\ v\left(x,y,z\right)=v_{1}\left(x,y\right)-z\frac{\partial w_{1}}{\partial y}-f\left(z\right)\frac{\partial w_{2}}{\partial y}\\ w\left(x,y,z\right)=w_{1}\left(x,y\right)+w_{2}\left(x,y\right) \end{array}\right.$$

where $u_{1}$ and $v_{1}$ are the tensile parts in the *x* and *y* directions, respectively. $w_{1}$ and $w_{2}$ are the bending component and shearing component, respectively. $f\left(z\right)$ is the shape function of *z*, and $f\left(z\right)=z-\varphi \left(z\right)$. $\varphi \left(z\right)$ adopts the shape function form proposed by Reissner, Reddy, and Touratier [33,34,35]. They can be given by:

(4) $$\mathrm{Reissner}:\;\varphi \left(z\right)=\frac{5z}{4}\left(1-\frac{4z^{2}}{3H^{2}}\right),\;\mathrm{Reddy}:\;\varphi \left(z\right)=z\left(1-\frac{4z^{2}}{3H^{2}}\right),\;\mathrm{Touratier}:\;\varphi \left(z\right)=\frac{H}{\pi }\sin\left(\frac{\pi z}{H}\right)$$

The shape function in this work defaults to the shape function proposed by Reissner.

The relationship between the strain and displacement fields is given by:

(5) $$\varepsilon_{xx}=\frac{\partial u}{\partial x},\;\varepsilon_{yy}=\frac{\partial v}{\partial y},\;\varepsilon_{zz}=\frac{\partial w}{\partial z},\;\gamma_{xy}=\frac{\partial v}{\partial x}+\frac{\partial u}{\partial y},\;\gamma_{yz}=\frac{\partial w}{\partial y}+\frac{\partial v}{\partial z},\;\gamma_{xz}=\frac{\partial w}{\partial x}+\frac{\partial u}{\partial z}$$

Substituting Equation (3) into Equation (5) gives:

(6) $$\left(\begin{array}{c} \varepsilon_{xx}\\ \varepsilon_{yy}\\ \gamma_{xy} \end{array}\right)=\left(\begin{array}{c} \varepsilon_{xx}^{1}\\ \varepsilon_{yy}^{1}\\ \gamma_{xy}^{1} \end{array}\right)+z\left(\begin{array}{c} \kappa_{xx}^{1}\\ \kappa_{yy}^{1}\\ \kappa_{xy}^{1} \end{array}\right)+f\left(z\right)\left(\begin{array}{c} \kappa_{xx}^{2}\\ \kappa_{yy}^{2}\\ \kappa_{xy}^{2} \end{array}\right),\;\varepsilon_{zz}=0,\;\left(\begin{array}{c} \gamma_{xz}\\ \gamma_{yz} \end{array}\right)=\left[1-f^{\prime }\left(z\right)\right]\left(\begin{array}{c} \gamma_{xz}^{2}\\ \gamma_{yz}^{2} \end{array}\right)$$

where:

(7) $$\left(\begin{array}{c} \varepsilon_{xx}^{1}\\ \varepsilon_{yy}^{1}\\ \gamma_{xy}^{1} \end{array}\right)=\left(\begin{array}{c} \frac{\partial u_{1}}{\partial x}\\ \frac{\partial v_{1}}{\partial y}\\ \frac{\partial u_{1}}{\partial y}+\frac{\partial v_{1}}{\partial x} \end{array}\right),\;\left(\begin{array}{c} \kappa_{xx}^{1}\\ \kappa_{yy}^{1}\\ \kappa_{xy}^{1} \end{array}\right)=-\left(\begin{array}{c} \frac{\partial^{2}w_{1}}{\partial x^{2}}\\ \frac{\partial^{2}w_{1}}{\partial y^{2}}\\ 2\frac{\partial^{2}w_{1}}{\partial x\partial y} \end{array}\right),\;\left(\begin{array}{c} \kappa_{xx}^{2}\\ \kappa_{yy}^{2}\\ \kappa_{xy}^{2} \end{array}\right)=-\left(\begin{array}{c} \frac{\partial^{2}w_{2}}{\partial x^{2}}\\ \frac{\partial^{2}w_{2}}{\partial y^{2}}\\ 2\frac{\partial^{2}w_{2}}{\partial x\partial y} \end{array}\right),\;\left(\begin{array}{c} \gamma_{xz}^{2}\\ \gamma_{yz}^{2} \end{array}\right)=\left(\begin{array}{c} \frac{\partial w_{2}}{\partial x}\\ \frac{\partial w_{2}}{\partial y} \end{array}\right)$$

According to the above strain field, the stress field of the FGM sandwich plate can be obtained using the constitutive relationship:

(8) $${\left(\begin{array}{c} \sigma_{xx}\\ \sigma_{yy}\\ \tau_{yz}\\ \tau_{xz}\\ \tau_{xy} \end{array}\right)}^{\left(i\right)}={\left[\begin{array}{ccccc} R_{11} & R_{12} & 0 & 0 & 0\\ R_{12} & R_{22} & 0 & 0 & 0\\ 0 & 0 & R_{44} & 0 & 0\\ 0 & 0 & 0 & R_{55} & 0\\ 0 & 0 & 0 & 0 & R_{66} \end{array}\right]}^{\left(i\right)}{\left(\begin{array}{c} \varepsilon_{xx}-\alpha T\\ \varepsilon_{yy}-\alpha T\\ \gamma_{yz}\\ \gamma_{xz}\\ \gamma_{xy} \end{array}\right)}^{\left(i\right)}\left(i=1,2,3\right)$$

where $R_{11},\;R_{12},\;R_{22},\;R_{44},\;R_{55},\;R_{66}$ can be expressed as:

(9) $$R_{11}^{\left(i\right)}=R_{22}^{\left(i\right)}=\frac{E^{\left(i\right)}\left(z\right)}{1-{\left(\vartheta^{\left(i\right)}\right)}^{2}},\;R_{12}^{\left(i\right)}=\vartheta^{\left(i\right)}R_{11}^{\left(i\right)},\;R_{44}^{\left(i\right)}=R_{55}^{\left(i\right)}=R_{66}^{\left(i\right)}=\frac{E^{\left(i\right)}\left(z\right)}{2\left(1+\vartheta^{\left(i\right)}\right)}$$

The total strain potential energy of the FGM sandwich plate is [36]:

(10) $$U=\frac{1}{2}\int_{V}\left[\sigma_{xx}^{\left(i\right)}{\left(\varepsilon_{xx}-\alpha T\right)}^{\left(i\right)}+\sigma_{yy}^{\left(i\right)}{\left(\varepsilon_{yy}-\alpha T\right)}^{\left(i\right)}+\tau_{xy}^{\left(i\right)}\gamma_{xy}^{\left(i\right)}+\tau_{xz}^{\left(i\right)}\gamma_{xz}^{\left(i\right)}+\tau_{yz}^{\left(i\right)}\gamma_{yz}^{\left(i\right)}\right]dV,\;\left(i=1,2,3\right)$$

where *V* is the volume of the FGM sandwich plate.

The external force is defined by:

(11) $$W=\int_{\Omega }qwd\Omega$$

where Ω is the top surface of the FGM sandwich plate.

The variational forms of Equations (10) and (11) are expressed as:

(12) $$\delta U=\int_{V}\left[\sigma_{xx}^{\left(i\right)}\delta \varepsilon_{xx}^{\left(i\right)}+\sigma_{yy}^{\left(i\right)}\delta \varepsilon_{yy}^{\left(i\right)}+\tau_{xy}^{\left(i\right)}\delta \gamma_{xy}^{\left(i\right)}+\tau_{xz}^{\left(i\right)}\delta \gamma_{xz}^{\left(i\right)}+\tau_{yz}^{\left(i\right)}\delta \gamma_{yz}^{\left(i\right)}\right]dV\left(i=1,2,3\right),\;\delta W=\int_{\Omega }q\delta wd\Omega$$

According to the principle of virtual work, one obtains:

(13) $$\int_{V}\left[\sigma_{xx}^{\left(i\right)}\delta \varepsilon_{xx}^{\left(i\right)}+\sigma_{yy}^{\left(i\right)}\delta \varepsilon_{yy}^{\left(i\right)}+\tau_{xy}^{\left(i\right)}\delta \gamma_{xy}^{\left(i\right)}+\tau_{xz}^{\left(i\right)}\delta \gamma_{xz}^{\left(i\right)}+\tau_{yz}^{\left(i\right)}\delta \gamma_{yz}^{\left(i\right)}\right]dV-\int_{\Omega }q\delta wd\Omega =0$$

Substituting Equation (6) and Equation (8) into Equation (13) and integrating *z*, Equation (13) can be rewritten as:

(14) $$\begin{array}{l} \int_{\Omega }[N_{xx}\delta \varepsilon_{xx}^{1}+N_{yy}\delta \varepsilon_{yy}^{1}+N_{xy}\delta \gamma_{xy}^{1}+M_{xx}^{1}\delta \kappa_{xx}^{1}+M_{yy}^{1}\delta \kappa_{yy}^{1}+M_{xy}^{1}\delta \kappa_{xy}^{1}+M_{xx}^{2}\delta \kappa_{xx}^{2}\\ +M_{yy}^{2}\delta \kappa_{yy}^{2}+M_{xy}^{2}\delta \kappa_{xy}^{2}+Q_{xz}^{2}\delta \kappa_{xz}^{2}+Q_{yz}^{2}\delta \kappa_{yz}^{2}]d\Omega -\int_{\Omega }q\delta wd\Omega =0 \end{array}$$

where $N_{xx},\;N_{yy},\;N_{xy},\;M_{xx}^{1},\;M_{yy}^{1},\;M_{xy}^{1},\;M_{xx}^{2},\;M_{yy}^{2},\;M_{xy}^{2},\;Q_{xz}^{2},\;Q_{yz}^{2}$ can be given by:

(15) $$\left[\begin{array}{ccc} N_{xx} & N_{yy} & N_{xy}\\ M_{xx}^{1} & M_{yy}^{1} & M_{xy}^{1}\\ M_{xx}^{2} & M_{yy}^{2} & M_{xy}^{2} \end{array}\right]=\overset{3}{\underset{i=1}{\sum }}\int_{z_{i}}^{z_{i+1}}\left(\begin{array}{c} 1\\ z\\ f\left(z\right) \end{array}\right){\left(\begin{array}{ccc} \sigma_{xx} & \sigma_{yy} & \sigma_{xy} \end{array}\right)}^{\left(i\right)}dz,\;\left[\begin{array}{c} Q_{xz}^{2}\\ Q_{yz}^{2} \end{array}\right]=\overset{3}{\underset{i=1}{\sum }}\int_{z_{i}}^{z_{i+1}}\left[1-f^{\prime }\left(z\right)\right]{\left(\begin{array}{c} \tau_{xz}\\ \tau_{yz} \end{array}\right)}^{\left(i\right)}dz$$

Substituting Equation (7) into Equation (14) and integrating by parts, and then letting the coefficients before $\delta u_{1}$, $\delta v_{1}$, $\delta w_{1}$, and $\delta w_{2}$ be zero, the following differential equation can be obtained as:

(16) $$\begin{array}{l} \delta u_{1}:\frac{\partial N_{xx}}{\partial x}+\frac{\partial N_{xy}}{\partial y}=0,\;\delta v_{1}:\frac{\partial N_{xy}}{\partial x}+\frac{\partial N_{yy}}{\partial y}=0,\;\delta w_{1}:\frac{\partial^{2}M_{xx}^{1}}{\partial x^{2}}+2\frac{\partial^{2}M_{xy}^{1}}{\partial x\partial y}+\frac{\partial^{2}M_{yy}^{1}}{\partial y^{2}}+q=0\\ \delta w_{2}:\frac{\partial^{2}M_{xx}^{2}}{\partial x^{2}}+2\frac{\partial^{2}M_{xy}^{2}}{\partial x\partial y}+\frac{\partial^{2}M_{yy}^{2}}{\partial y^{2}}+\frac{\partial Q_{xz}^{2}}{\partial x}+\frac{\partial Q_{yz}^{2}}{\partial y}+q=0 \end{array}$$

Substituting Equations (6) and (8) into Equation (15) gives:

(17) $$\left(\begin{array}{c} N\\ M^{1}\\ M^{2} \end{array}\right)=\left[\begin{array}{ccc} A & A^{1} & B^{1}\\ A^{1} & B & C^{1}\\ B^{1} & C^{1} & C \end{array}\right]\left(\begin{array}{c} \varepsilon^{1}\\ \kappa^{1}\\ \kappa^{2} \end{array}\right)-\left(\begin{array}{c} N^{t}\\ M^{1t}\\ M^{2t} \end{array}\right),\;\left[\begin{array}{c} Q_{yz}^{2}\\ Q_{xz}^{2} \end{array}\right]=\left[\begin{array}{cc} E_{44} & 0\\ 0 & E_{55} \end{array}\right]\left(\begin{array}{c} \gamma_{yz}^{2}\\ \gamma_{xz}^{2} \end{array}\right)$$

where:

(18) $$\begin{array}{l} \left(N,\;M^{1},\;M^{2}{)}^{T}=(N_{xx},\;N_{yy},\;N_{xy},\;M_{xx}^{1},\;M_{yy}^{1},\;M_{xy}^{1},\;M_{xx}^{2},\;M_{yy}^{2},\;M_{xy}^{2}\right)\\ {\left(N^{t},\;M^{1t},\;M^{2t}\right)}^{T}={\left(N_{xx}^{t},\;N_{yy}^{t},\;0,\;M_{xx}^{1t},\;M_{yy}^{1t},\;0,\;M_{xx}^{2t},\;M_{yy}^{2t},\;0\right)}^{T}\\ {\left(\varepsilon^{1},\;\kappa^{1},\;\kappa^{2}\right)}^{T}=(\varepsilon_{xx}^{1},\;\varepsilon_{yy}^{1},\;\gamma_{xy}^{1},\;\kappa_{xx}^{1},\;\kappa_{yy}^{1},\;\kappa_{xy}^{1},\;\kappa_{xx}^{2},\;\kappa_{yy}^{2},\;\kappa_{xy}^{2}{)}^{T} \end{array}$$

and:

(19) $$\begin{array}{l} A=\left(\begin{array}{ccc} A_{11} & A_{12} & 0\\ A_{12} & A_{22} & 0\\ 0 & 0 & A_{66} \end{array}\right),\;A^{1}=\left(\begin{array}{ccc} A_{11}^{1} & A_{12}^{1} & 0\\ A_{12}^{1} & A_{22}^{1} & 0\\ 0 & 0 & A_{66}^{1} \end{array}\right),\;B=\left(\begin{array}{ccc} B_{11} & B_{12} & 0\\ B_{12} & B_{22} & 0\\ 0 & 0 & B_{66} \end{array}\right)\\ B^{1}=\left(\begin{array}{ccc} B_{11}^{1} & B_{12}^{1} & 0\\ B_{12}^{1} & B_{22}^{1} & 0\\ 0 & 0 & B_{66}^{1} \end{array}\right),\;C=\left(\begin{array}{ccc} C_{11} & C_{12} & 0\\ C_{12} & C_{22} & 0\\ 0 & 0 & C_{66} \end{array}\right),\;C^{1}=\left(\begin{array}{ccc} C_{11}^{1} & C_{12}^{1} & 0\\ C_{12}^{1} & C_{22}^{1} & 0\\ 0 & 0 & C_{66}^{1} \end{array}\right) \end{array}$$

The specific forms of elements in matrices $A,\;A^{1},\;B,\;B^{1},\;C,\;C^{1}$, and $E_{44},\;E_{55}$ can be written as:

(20) $$\begin{array}{l} \left(\begin{array}{c} A_{11}\\ A_{12}\\ A_{66} \end{array}\begin{array}{c} A_{11}^{1}\\ A_{12}^{1}\\ A_{66}^{1} \end{array}\begin{array}{c} B_{11}\\ B_{12}\\ B_{66} \end{array}\begin{array}{c} B_{11}^{1}\\ B_{12}^{1}\\ B_{66}^{1} \end{array}\begin{array}{c} C_{11}\\ C_{12}\\ C_{66} \end{array}\begin{array}{c} C_{11}^{1}\\ C_{12}^{1}\\ C_{66}^{1} \end{array}\right)=\sum_{i=1}^{3}\int_{z_{i}}^{z_{i+1}}\left(\begin{array}{c} R_{11}^{\left(i\right)}\\ R_{12}^{\left(i\right)}\\ R_{66}^{\left(i\right)} \end{array}\right)\left(\begin{array}{cccc} \begin{array}{cc} \begin{array}{cc} 1 & z \end{array} & z^{2} \end{array} & f\left(z\right) & zf\left(z\right) & f^{2}\left(z\right) \end{array}\right)dz\\ \left(\begin{array}{c} E_{44}\\ E_{55} \end{array}\right)=\sum_{i=1}^{3}\int_{z_{i}}^{z_{i+1}}\left[1-f^{\prime }\left(z\right)\right]\left(\begin{array}{c} R_{44}^{\left(i\right)}\\ R_{55}^{\left(i\right)} \end{array}\right)dz \end{array}$$

The matrix elements related to thermal load in Equation (17) are $N_{xx}^{t}$, $N_{yy}^{t}$, $M_{xx}^{1t}$, $M_{yy}^{1t}$, $M_{xx}^{2t}$, and $M_{yy}^{2t}$. They can be written as:

(21) $$\left[\begin{array}{ccc} N_{xx}^{t} & M_{xx}^{1t} & M_{xx}^{2t}\\ N_{yy}^{t} & M_{yy}^{1t} & M_{yy}^{2t} \end{array}\right]=\overset{3}{\underset{i=1}{\sum }}\int_{z_{i}}^{z_{i+1}}{\left(\begin{array}{c} \left(R_{11}+R_{12}\right)\alpha T\\ (R_{12}+R_{22})\alpha T \end{array}\right)}^{\left(i\right)}\left(\begin{array}{ccc} 1 & z & f\left(z\right) \end{array}\right)dz$$

For the temperature field *T*, the nonlinear temperature field that varies along the thickness of the plate used by Mantari [37] is adopted in the paper, and the specific form is as follows:

(22) $$T\left(x,y,z\right)=T_{1}\left(x,y\right)+\frac{z}{H}T_{2}\left(x,y\right)+\frac{\varphi \left(z\right)}{H}T_{3}\left(x,y\right)$$

where $T_{1}\left(x,y\right)$ is the temperature field that does not change in the thickness direction, $T_{2}\left(x,y\right)$ is the temperature field that changes linearly, and $T_{3}\left(x,y\right)$ is the temperature field that changes nonlinearly.

Under the simply supported boundary condition, the following relations are obtained:

(23) $$\begin{array}{c} x=0,\;L_{1}:\;v_{1}=w_{1}=w_{2}=0,\;\frac{\partial w_{1}}{\partial y}=\frac{\partial w_{2}}{\partial y}=0,\;N_{xx}=0,\;M_{xx}^{1}=M_{xx}^{2}=0\\ y=0,\;L_{2}:\;u_{1}=w_{1}=w_{2}=0,\;\frac{\partial w_{1}}{\partial x}=\frac{\partial w_{2}}{\partial x}=0,\;N_{yy}=0,\;M_{yy}^{1}=M_{yy}^{2}=0 \end{array}$$

To solve the above model, the Navier method is used in this paper, and the following assumptions are made for bi-sinusoidal load, temperature field, and displacement field:

(24) $$q=q_{0}\sin(mx)\sin(ny),\;\left(\begin{array}{c} T_{1}\\ T_{2}\\ T_{3} \end{array}\right)=\left(\begin{array}{c} t_{1}\\ t_{2}\\ t_{3} \end{array}\right)\sin\left(mx\right)\sin\left(ny\right),\;\left(\begin{array}{c} u_{1}\\ v_{1}\\ w_{1}\\ w_{2} \end{array}\right)=\left(\begin{array}{c} U\cos\left(mx\right)\sin(ny)\\ V\sin\left(mx\right)\cos\left(ny\right)\\ W_{1}\sin\left(mx\right)\sin\left(ny\right)\\ W_{2}\sin\left(mx\right)\sin\left(ny\right) \end{array}\right)$$

where $q_{0},\;t_{1},\;t_{2},\;t_{3},\;U,\;V,\;W_{1},\;W_{2}$ are constants. $m=\pi /L_{1}$ and $n=\pi /L_{2}$.

Based on the above assumptions, the following operator equation can be obtained:

(25) $$\left[\Gamma \right]\left[\Lambda \right]=\left[F\right]$$

where $\left[\Gamma \right]$ denotes the stiffness coefficient matrix, and $\left[F\right]$ denotes the displacement vector and the generalized force. The displacement vector $\left[\Lambda \right]$ is ${\left(\begin{array}{cccc} U & V & W_{1} & W_{2} \end{array}\right)}^{T}$. For the stiffness coefficient matrix $\left[\Gamma \right]$ and the generalized force $\left[F\right]$ see Appendix A.

## 3. Model Validation and Numerical Analysis

In this part, the numerical example of a 1-1-1 FGM sandwich plate is given and discussed to verify the accuracy of the present method in predicting the bending of simply supported FGM sandwich plates under thermo-mechanical loads. In addition, several numerical examples of thermo-mechanical bending of asymmetrical FGM sandwich plates under simply supported boundary conditions are also given and analyzed.

The FGM is composed of titanium alloy (as metal) and zirconia (as ceramic). Their material properties [38] are listed in Table 1.

Unless mentioned otherwise, the following properties are used:

(26) $$L_{1}/H=10,\;L_{1}=L_{2},\;q_{0}=100,\;t_{1}=0,\;t_{2}=t_{3}=100K$$

The dimensionless deflection and stress are defined as:

(27) $$\begin{array}{c} \bar{w}=\frac{10^{3}}{q_{0}L_{1}^{4}/(E_{0}H^{3})+10^{3}\alpha_{0}t_{2}L_{1}^{2}/H}w\left(\frac{L_{1}}{2},\frac{L_{2}}{2}\right),\;{\bar{\sigma }}_{xx}=\frac{10^{3}}{q_{0}L_{1}^{2}/H^{2}+10E_{0}\alpha_{0}t_{2}L_{1}^{2}/H^{2}}\sigma_{xx}\left(\frac{L_{1}}{2},\frac{L_{2}}{2},\frac{H}{2}\right)\\ {\bar{\tau }}_{xz}=\frac{10^{3}}{q_{0}L_{1}/H+E_{0}\alpha_{0}t_{2}L_{1}/(10H)}\tau_{xz}\left(0,\frac{L_{2}}{2},0\right) \end{array}$$

where $E_{0}=1\mathrm{Gpa},\;\alpha_{0}=10^{-6}/K$.

The sandwich structure is represented by the ratio of the thickness of each layer. This article uses five types of sandwich structures: 1-1-1, 1-1-2, 1-2-2, 1-2-3, and 2-1-3. For example, 1-1-1 represents $z_{1}=-H/2,\;z_{2}=-H/6,\;z_{3}=H/6,\;z_{4}=H/2$, 1-1-2 represents $z_{1}=-H/2,\;z_{2}=0,\;z_{3}=H/4,\;z_{4}=H/2$, etc.

### 3.1. Model Validation

In order to verify the accuracy of the model in this paper, the dimensionless natural deflections and stresses of 1-1-1 FGM sandwich plates calculated according to the model in this paper under the simply supported condition are compared with the theoretical results of the sinusoidal shear deformation plate theory (SSDPT), the third-order shear deformation plate theory (TSDPT), and the first-order shear deformation plate theory (FSDPT). The results are shown in Table 2.

It can be seen from Table 2 that the results of this paper are in good agreement with the results of the literature, which verifies the correctness of the model. In addition, $\bar{w}$ of 1-1-1 FGM sandwich plates increases with the value of s, and dimensionless stresses of 1-1-1 FGM sandwich plates decrease with the increase in the value of s (s>0).

### 3.2. Parameter Study

In order to study the effects of side-to-thickness ratio $L_{1}/H$, volume fraction index s, and nonlinear temperature $t_{3}$ on the deflections and stresses of asymmetric FGM sandwich plates under simply supported boundary conditions, parameter studies are carried out in this section.

Table 3 and Table 4 show the dimensionless deflection and stress of four types of asymmetric FGM sandwich plates under different shape functions at s=0,1,3,5.

It can be seen from Table 3 and Table 4 that for a given value of s and layer thickness ratio, dimensionless center deflection and dimensionless normal stress calculated by the Reissener method are the largest, whereas dimensionless center deflection and dimensionless normal stress calculated by the Touratier method are the smallest. In addition, for a given layer thickness ratio, $\bar{w}$ increases with the value of s, while ${\bar{\sigma }}_{xx}$ decreases with the increase in the value of s (s>0).

**Figure 2:**
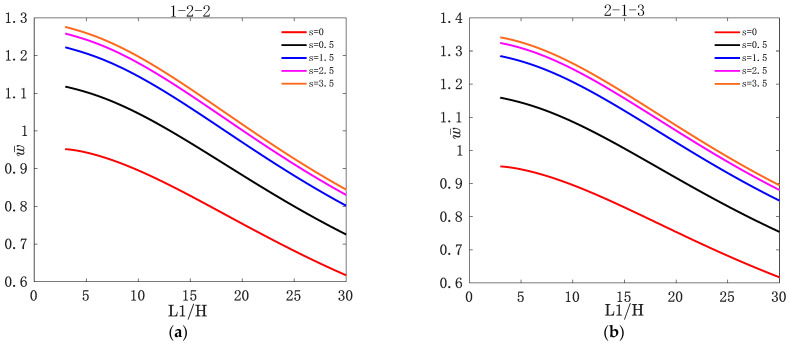
Variation of dimensionless deflection $\bar{w}$ with $L_{1}/H$ for two types of asymmetric FGM sandwich plate under different volume fraction indices s: (**a**) 1-2-2; (**b**) 2-1-3.

**Figure 3:**
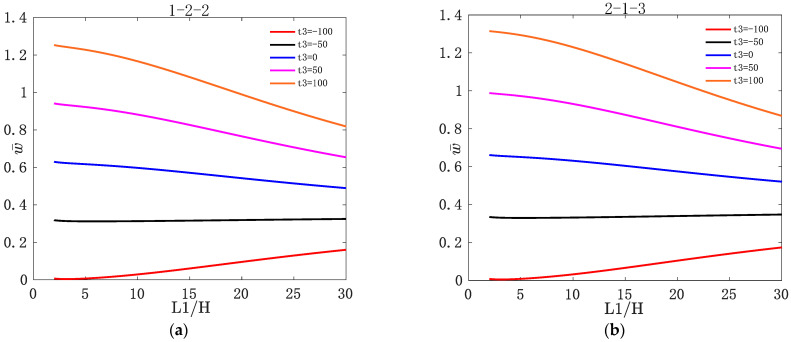
Variation of dimensionless deflection $\bar{w}$ with $L_{1}/H$ for two types of asymmetric FGM sandwich plate under different nonlinear temperatures $t_{3}$: (**a**) 1-2-2; (**b**) 2-1-3.

**Figure 4:**
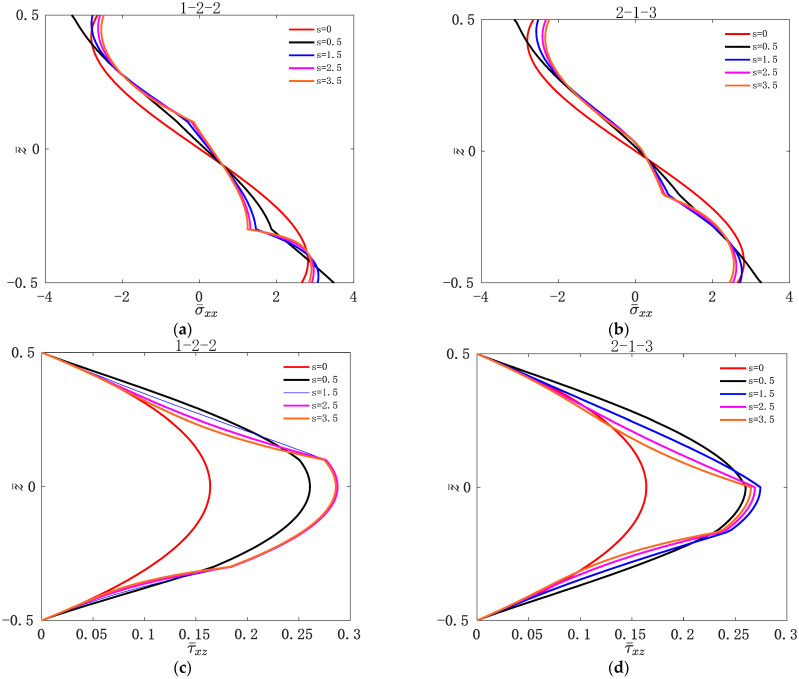
Variation of dimensionless stress ${\bar{\sigma }}_{xx}$ and ${\bar{\tau }}_{xz}$ with $\bar{z}$ for two types of asymmetric FGM sandwich plate under different volume fraction indices s: (**a**) ${\bar{\sigma }}_{xx}$ of 1-2-2; (**b**) ${\bar{\sigma }}_{xx}$ of 2-1-3; (**c**) ${\bar{\tau }}_{xz}$ of 1-2-2; (**d**) ${\bar{\tau }}_{xz}$ of 2-1-3.

**Figure 5:**
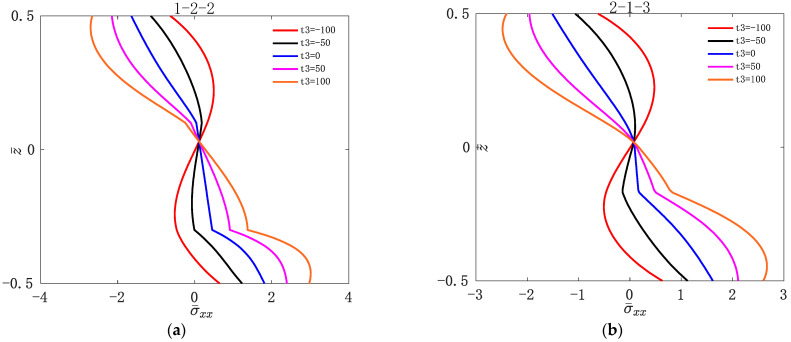

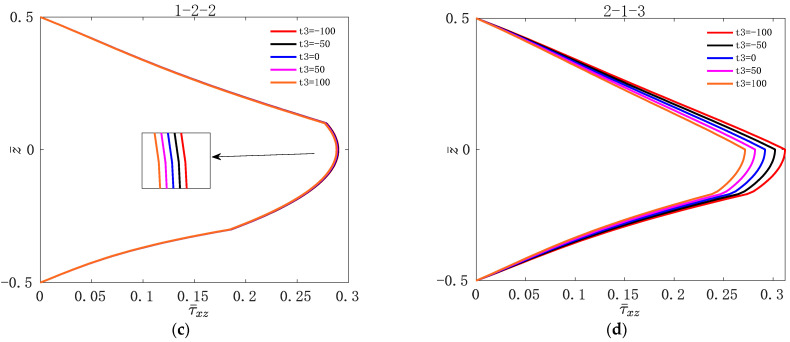
Variation of dimensionless stress ${\bar{\sigma }}_{xx}$ and ${\bar{\tau }}_{xz}$ with $\bar{z}$ for two types of asymmetric FGM sandwich plate under different nonlinear temperatures $t_{3}$: (**a**) ${\bar{\sigma }}_{xx}$ of 1-2-2; (**b**) ${\bar{\sigma }}_{xx}$ of 2-1-3; (**c**) ${\bar{\tau }}_{xz}$ of 1-2-2; (**d**) ${\bar{\tau }}_{xz}$ of 2-1-3.

Figure 2 shows the variation of $\bar{w}$ with $L_{1}/H$ for two types of asymmetric FGM sandwich plate under different values of s. Figure 3 shows the variation of $\bar{w}$ with $L_{1}/H$ for two types of asymmetric FGM sandwich plate under different values of $t_{3}$. Figure 4 shows the variation of ${\bar{\sigma }}_{xx}$ and ${\bar{\tau }}_{xz}$ with $\bar{z}$ for two types of asymmetric FGM sandwich plate under different values of s. Figure 5 shows the variation of ${\bar{\sigma }}_{xx}$ and ${\bar{\tau }}_{xz}$ with $\bar{z}$ for two types of asymmetric FGM sandwich plate under different values of $t_{3}$.

It can be seen from Figure 2 that $\bar{w}$ of the asymmetric FGM sandwich plate decreases with the increase in $L_{1}/H$. This is because for asymmetric FGM sandwich plates with simply supported boundary conditions, the larger the side-to-thickness ratio, the greater the stiffness of the sandwich plate, resulting in a decrease in its deflection. For a certain layer thickness ratio, $\bar{w}$ of the asymmetric FGM sandwich plate increases with the value of s. This is because the ceramic volume content of the asymmetric FGM sandwich plates decreases with the increase in the volume fraction index s, resulting in a decrease in the stiffness of the sandwich plate, and an increase in the deflection of the sandwich plate with the increase in volume fraction index s.

It can be seen from Figure 3 that when $t_{3}>0$, $\bar{w}$ of asymmetric FGM sandwich plates decreases with the increase in $L_{1}/H$. However, when $t_{3}<0$, $\bar{w}$ of asymmetric FGM sandwich plates increases with $L_{1}/H$. For a certain layer thickness ratio, $\bar{w}$ of asymmetric FGM sandwich plates increases with the value of $t_{3}$. This is because as the temperature increases, the stiffness of the asymmetric FGM sandwich plate decreases, resulting in a decrease in the deflection of the sandwich plate as the temperature increases.

For sandwich plates, with the top plate subjected to bi-sinusoidal loads, dimensionless positive stresses are compressive stresses above the middle plane and tensile stresses below the middle plane.

It can be seen from Figure 4 that the stress is continuously distributed along the thickness direction. Regardless of the value of s, the maximum compressive stress and the maximum tensile stress are on the upper and lower layers of the asymmetric FGM sandwich plate, respectively. This is because during the upward bending process of both ends of the asymmetric FGM sandwich plates, the compressive stress increases continuously above the middle plane, and the tensile stress increases continuously below the middle plane. The maximum shear stress occurs in the core of the asymmetric FGM sandwich plate. This is because there will be an increase in shear force during the core layer, which results in the maximum shear stress appearing in the core layer.

It can be seen from Figure 5 that ${\bar{\sigma }}_{xx}$ and ${\bar{\tau }}_{xz}$ of the asymmetric FGM sandwich plates are all sensitive to $t_{3}$. No matter how large the value of $t_{3}$ is, the maximum compressive stress and the maximum tensile stress are on the upper and lower layers of the sandwich plate, respectively, and the maximum of ${\bar{\tau }}_{xz}$ occurs in the core layer of the asymmetric FGM sandwich plate. ${\bar{\tau }}_{xz}$ decreases gradually with the increase in the value of $t_{3}$. This is because the shear force decreases with the increase in temperature, which in turn affects the shear stress.

## 4. Conclusions

In this study, the refined shear deformation theory is extended to the thermo-mechanical bending analysis of asymmetric rectangular FGM sandwich plates. Based on the principle of virtual work, its governing equation is obtained, and its solution under simply supported boundary conditions is obtained using the Navier method. To verify the accuracy of the theory presented in this paper, the bending results of symmetric FGM sandwich plates under thermo-mechanical loading are compared with those in other research. Finally, the effects of the volume fraction index, geometric ratio, layer thickness ratio, and nonlinear temperature on the deflection and stress of asymmetric functionally graded material sandwich plates are investigated. The following conclusions are reached:

1. In contrast with other theories, this theory only generates four control equations and only four unknown variables are involved in solving the control equation. The complexity and workload of calculation are significantly reduced.
2. By comparing the results of 1-1-1 FGM sandwich plates with those published in the literature, it can be seen that the theoretical model in this paper is accurate in predicting the bending performance of FGM sandwich plates under thermo-mechanical load.
3. For the asymmetric FGM sandwich plate, the stress is continuous, but not smooth, especially at the interface.
4. For asymmetric FGM sandwich plates, regardless of the volume fraction index, layer thickness ratio, and nonlinear temperature, the maximum compressive stress is always generated on the top plate, the maximum tensile stress is always generated on the bottom plate, and maximum shear stress always occurs in the core layer.

## Figures and Tables

**Table 1:** Material properties of metals and ceramics in FGM.

	Ti-6Al-4V	ZrO_2_
Young’s modulus (GPa)	66.2	117.0
Poisson’s ratio	1/3	1/3
Thermal expansion coefficient (10^−6^/K)	10.3	7.11

**Table 2:** Dimensionless deflections and stresses of 1-1-1 FGM sandwich plates.

*S*	Theory	$\bar{w}$	${\bar{\sigma }}_{xx}$	${\bar{\tau }}_{xz}$
0	SSDPT [38]	0.796783	−2.388909	0.171603
	TSDPT [15]	0.808168	−2.461177	0.174481
	FSDPT [11]	0.895735	−3.597007	0.173624
	Present	0.895427	−2.650327	0.163882
1	SSDPT [38]	1.011263	−2.659816	0.289195
	TSDPT [15]	1.025367	−2.730494	0.280495
	FSDPT [11]	1.132449	−3.756017	0.203004
	Present	1.136261	−2.961917	0.280083
3	SSDPT [38]	1.092312	−2.262512	0.282953
	TSDPT [15]	1.107475	−2.328042	0.276238
	FSDPT [11]	1.223232	−3.311823	0.221768
	Present	1.227011	−2.517070	0.273899
5	SSDPT [38]	1.112660	−2.162596	0.273950
	TSDPT [15]	1.128152	−2.226550	0.269077
	FSDPT [11]	1.246833	−3.196423	0.228818
	Present	1.249763	−2.405376	0.265250

**Table 3:** Dimensionless center deflections $\bar{w}$ of the different asymmetric FGM sandwich plates.

*s*	Theory	$\bar{w}$			
		1-1-2	1-2-2	1-2-3	2-1-3
0	Reissener	0.895427	0.895427	0.895427	0.895427
	Reddy	0.808168	0.801678	0.808168	0.808168
	Touratier	0.796783	0.796783	0.796783	0.796783
1	Reissener	1.142143	1.110215	1.112215	1.165239
	Reddy	1.030702	1.001863	1.003709	1.051579
	Touratier	1.016519	0.988059	0.989855	1.037101
3	Reissener	1.226837	1.190182	1.185903	1.255485
	Reddy	1.107398	1.074180	1.070394	1.133342
	Touratier	1.092173	1.059464	1.055662	1.117730
5	Reissener	1.245792	1.210657	1.202900	1.274230
	Reddy	1.124634	1.092766	1.085831	1.150416
	Touratier	1.109130	1.077772	1.070861	1.134501

**Table 4:** Dimensionless normal stress ${\bar{\sigma }}_{xx}$ of the different asymmetric FGM sandwich plates.

*s*	Theory	${\bar{\sigma }}_{xx}$			
		1-1-2	1-2-2	1-2-3	2-1-3
0	Reissener	−2.650327	−2.650327	−2.650327	−2.650327
	Reddy	−2.461177	−2.461177	−2.461177	−2.461177
	Touratier	−2.388909	−2.388909	−2.388909	−2.388909
1	Reissener	−3.066004	−2.967628	−2.890075	−2.738889
	Reddy	−2.824426	−2.735543	−2.665281	−2.528809
	Touratier	−2.752510	−2.664883	−2.595690	−2.460966
3	Reissener	−2.693963	−2.527080	−2.461049	−2.275796
	Reddy	−2.488009	−2.336886	−2.276629	−2.109048
	Touratier	−2.420248	−2.271451	−2.212478	−2.047177
5	Reissener	−2.606560	−2.414872	−2.364258	−2.184386
	Reddy	−2.408708	−2.234923	−2.188565	−2.025707
	Touratier	−2.342244	−2.177731	−2.540488	−1.965394

## Data Availability

Data sharing not applicable.

## Appendix A

The stiffness coefficient matrix Γ is given by:

(A1) $$\begin{array}{l} \Gamma_{11}=A_{11}m^{2}+A_{66}n^{2},\;\Gamma_{12}=mn\left(A_{12}+A_{66}\right),\;\Gamma_{13}=-m\left[A_{11}^{1}m^{2}+\left(A_{12}^{1}+2A_{66}^{1}\right)n^{2}\right]\\ \Gamma_{14}=-m\left[B_{11}^{1}m^{2}+\left(B_{12}^{1}+2B_{66}^{1}\right)n^{2}\right],\;\Gamma_{22}=A_{66}m^{2}+A_{66}n^{2},\;\Gamma_{23}=-n\left[A_{22}^{1}n^{2}+\left(A_{12}^{1}+2A_{66}^{1}\right)m^{2}\right]\\ \Gamma_{24}=-n\left[B_{22}^{1}n^{2}+\left(B_{12}^{1}+2B_{66}^{1}\right)m^{2}\right],\;\Gamma_{33}=B_{11}m^{4}+2\left(B_{12}+2B_{66}\right)m^{2}n^{2}+B_{22}n^{4}\\ \Gamma_{34}=C_{11}^{1}m^{4}+2\left(C_{12}^{1}+2C_{66}^{1}\right)m^{2}n^{2}+C_{22}^{1}n^{4},\;\Gamma_{44}=C_{11}m^{4}+2\left(C_{12}+2C_{66}\right)m^{2}n^{2}+C_{22}n^{4}+E_{44}n^{4}+E_{55}m^{4} \end{array}$$

The generalized force vector $\left[F\right]={\left[\begin{array}{cccc} F_{1} & F_{2} & F_{3} & F_{4} \end{array}\right]}^{T}$ can be written as:

(A2) $$\begin{array}{l} F_{1}=-m\left(A_{T}t_{1}+B_{T}t_{2}+B_{T}^{a}t_{3}\right),\;F_{2}=-n\left(A_{T}t_{1}+B_{T}t_{2}+B_{T}^{a}t_{3}\right)\\ F_{3}=q_{0}+h\left(m^{2}+n^{2}\right)\left(B_{T}t_{1}+C_{T}t_{2}+C_{T}^{a}t_{3}\right),\;F_{4}=q_{0}+h\left(m^{2}+n^{2}\right)\left(D_{T}t_{1}+E_{T}t_{2}+E_{T}^{a}t_{3}\right) \end{array}$$

where:

(A3) $$\begin{array}{l} \left(\begin{array}{c} A_{T}\\ B_{T}\\ C_{T} \end{array}\right)=\sum_{i=1}^{3}\int_{z_{i}}^{z_{i+1}}\frac{E^{\left(i\right)}}{1-{\left(\vartheta^{\left(i\right)}\right)}^{2}}\left(1+\vartheta^{\left(i\right)}\right)\alpha^{\left(i\right)}\left(\begin{array}{c} 1\\ \bar{z}\\ {\bar{z}}^{2} \end{array}\right)dz,\left(\begin{array}{c} B_{T}^{a}\\ C_{T}^{a} \end{array}\right)=\sum_{i=1}^{3}\int_{z_{i}}^{z_{i+1}}\frac{E^{\left(i\right)}}{1-{\left(\vartheta^{\left(i\right)}\right)}^{2}}\left(1+\vartheta^{\left(i\right)}\right)\alpha^{\left(i\right)}\bar{\varphi }\left(z\right)\left(\begin{array}{c} 1\\ \bar{z} \end{array}\right)dz\\ \left(\begin{array}{c} D_{T}\\ E_{T}\\ E_{T}^{a} \end{array}\right)=\sum_{i=1}^{3}\int_{z_{i}}^{z_{i+1}}\frac{E^{\left(i\right)}}{1-{\left(\vartheta^{\left(i\right)}\right)}^{2}}\left(1+\vartheta^{\left(i\right)}\right)\alpha^{\left(i\right)}\bar{f}\left(z\right)\left(\begin{array}{c} 1\\ \bar{z}\\ \bar{\varphi }\left(z\right) \end{array}\right)dz \end{array}$$

in which $\bar{z}=\frac{z}{H},\;\bar{\varphi }\left(z\right)=\frac{\varphi \left(z\right)}{H},\;\bar{f}\left(z\right)=\frac{f\left(z\right)}{z}$.
